# AS04 drives superior cross-protective antibody response by increased NOTCH signaling of dendritic cells and proliferation of memory B cells

**DOI:** 10.3389/fimmu.2025.1623405

**Published:** 2025-07-24

**Authors:** Valentino D’Onofrio, Ana Carolina Santana, Marthe Pauwels, Gwenn Waerlop, Anthony Willems, Fien De Boever, Martin Müller, Peter Sehr, Tim Waterboer, Isabel Leroux-Roels, Ashish A. Sharma, Rafick Pierre Sékaly, Geert Leroux-Roels

**Affiliations:** ^1^ Department of Diagnostic Sciences, Ghent University and Ghent University Hospital, Ghent, Belgium; ^2^ Pathology Advanced Translational Research Unit, Department of Pathology and Laboratory Medicine, Emory University School of Medicine, Atlanta, GA, United States; ^3^ Emory Vaccine Center, Emory University School of Medicine, Atlanta, GA, United States; ^4^ Division of Infections and Cancer Epidemiology, German Cancer Research Center (DKFZ), Heidelberg, Germany; ^5^ Chemical Biology Core Facility, European Molecular Biology Laboratory (EMBL), Heidelberg, Germany

**Keywords:** HPV vaccine, adjuvant, immune response, dendritic cells, memory B cell

## Abstract

**Introduction:**

The Gardasil-4^®^ vaccine targets HPV types 6, 11, 16 and 18 and is formulated with amorphous alum. Cervarix^®^ targets HPV types 16 and 18 using AS04 (Al(OH)3 + TLR4 agonist MPL) to enhance immune response. Cervarix elicits higher cross-protection against other high-risk HPV types, likely mediated by AS04.

**Methods:**

To investigate mechanisms of cross-neutralizing potential, six monozygotic twins (12 females aged 9-13 years) were vaccinated with either Cervarix or Gardasil-4 (2 doses, 6 months apart). Serum neutralizing antibody titers against HPV 6,16,18,31,33,45,52, and 58 were assessed pre-vaccination and 7 days post-second dose. Multi-omic single cell RNA and ATAC sequencing of PBMCs was performed at the latter timepoint.

**Results:**

Cervarix generated higher cross-neutralizing antibody titers than Gardasil-4. Higher frequencies of dendritic cells and memory B cells were observed. Gene Set Enrichment Analysis (GSEA) indicated enhanced pathways related to NOTCH2 signaling in DCs and cell cycling/RNA translation in B cells, correlating positively with cross-neutralizing antibody titers. Increased chromatin accessability in genes related to NOTCH signaling in cDC1 was also observed. Cervarix-vaccinated subjects showed increased DC-to-memory B signaling, through upregulation of NOTCH ligands. Engagement of NOTCH was associated to BCL2 expression in memory B cells, supporting an anti-apoptotic state.

**Conclusion:**

Increased DC signaling, including NOTCH, through AS04 in Cervarix supports cell survival and sustained RNA translation in memory B cells, 7 days post-vaccination. This may enhance adaptive immune cell maturation, providing a mechanism that can lead to improved cross-reactivity.

## Introduction

Human papillomavirus (HPV) is one of the most prevalent sexually transmitted infections worldwide, causing cervical cancer and contributing to other malignancies, including vulvar, vaginal, anal, penile, and oropharyngeal cancers (1). Notably, over 99% of cervical cancer cases can be attributed to persistent infection with high-risk HPV types, particularly HPV16 and HPV18, which together account for approximately 70% of global cases (2). Additionally, non-oncogenic HPV types, such as HPV6 and HPV11, are associated with benign anogenital and non-genital warts, underscoring HPV infections as a significant public health concern (1, 3).

To address this significant health burden posed by HPV-related diseases, prophylactic HPV vaccines have been developed to prevent infection and associated malignancies (4). Currently, three globally licensed HPV vaccines are based on the self-assembly properties of the HPV-type specific major capsid protein L1, into virus-like particles (VLPs) (4). While all licensed vaccines are L1-containing VLPs, they differ in their antigen content and adjuvant formulations. Cervarix^®^ (GlaxoSmithKline Vaccines) is a bivalent vaccine targeting HPV16 and HPV18, and is formulated with the Adjuvant System 04 (AS04), comprising aluminum hydroxide salts (Al(OH)_3_) and the Toll-like receptor 4 (TLR4) agonist 3-O-desacyl-4’-monophosphoryl lipid A (MPL) (5). Gardasil-4^®^ (Merck) is a quadrivalent vaccine formulated with amorphous aluminum hydroxyphosphate sulfate (AlHO9PS-3) as its adjuvant, targeting HPV16, HPV18, and the low-risk types HPV6 and HPV11 (6). Gardasil-9^®^ (Merck) maintains the same aluminum-based adjuvant but extends Gardasil-4’s protection by including five additional high-risk HPV types (31, 33, 45, 52, and 58), which collectively account for approximately 20% of cervical cancers (7).

While HPV vaccines are highly effective and safe in preventing persistent HPV-infections and precancerous cervical lesions caused by high-risk HPV types (8, 9), Cervarix and Gardasil-4 have demonstrated differences in their immunological profiles (10–13). Both vaccines elicit high levels of neutralizing serum antibodies against vaccine-specific HPV types (14). However, Cervarix induces higher HPV16 and HPV18 antibody titers, greater frequencies of HPV16/18-specific memory B cells, and more robust antibody-dependent complement activation compared to Gardasil-4 (10, 15–17). Both vaccines confer a differential degree of cross-protection by inducing cross-protective neutralizing antibodies towards phylogenetically related, non-vaccine HPV types within the Alpha-papillomavirus species group A9 (HPV16-like: 31, 33, 35, 52, 58) and A7 (HPV18-like: 39, 45, 59, 68) (18). Cervarix elicits broader cross-neutralization capabilities and higher magnitudes of cross-protective antibodies towards HPV31, 33, 45, and 52, compared to Gardasil-4 (10, 13, 15, 17, 18). The superior cross-protective efficacy of Cervarix can be attributed to its AS04 adjuvant. In preclinical models, AS04 directly stimulates conventional dendritic cells upon TLR4 engagement, thereby enhancing antigen presentation and promoting T-cell activation, particularly Th1 CD4+ T cells and CD8+ T cells (19, 20). Compared to other adjuvant systems (AS01B, AS01E, and AS03), AS04 has been shown to induce weaker immune responses in terms of CD4+ T cell frequencies. While MPL activates TLR4 and induces a strong Th1 response, this effect is enhanced by the synergistic effect of QS-21 in AS01, possibly explaining the lower CD4+ T cell frequencies after AS04 compared to AS01 (21). Moreover, AS04 stimulates follicular helper T cells (Tfh) that interact with memory B cells, present in primed individuals, and stimulate them to rapidly produce high-affinity antibodies. Indeed, AS04 did show the capacity to not only boost antibody responses but also induce different antibody-effector functions, compared to alumn (19, 21). However, the complete molecular mechanisms underlying the broader cross-neutralization capacities of Cervarix remain to be further elucidated.

This study aimed to investigate the molecular mechanisms by which AS04 induces cross-protective antibodies against closely related HPV types not included in Cervarix, in comparison to Gardasil-4. In PBMCs collected seven days after the second vaccine dose, we confirmed an increased breadth of neutralizing antibodies. Additionally, we observed enhanced conventional Dendritic Cells type 1 (cDC1) signaling, including upregulated NOTCH signaling, as evidenced by increased gene expression and chromatin accessibility. This activation was associated with active cell proliferation seven days after the second dose, contributing to the improved maturation of adaptive immune cells.

## Methods

### Study design

A blinded randomized interventional study was performed at the Center for Vaccinology (CEVAC, Ghent University and Ghent University Hospital) in Ghent, Belgium between June 2014 and April 2015. Participants were homozygous female twins between 9 and 13 years that were eligible for HPV vaccination according to national recommendations. Twins were recruited from the East Flanders Prospective Twin Survey. All female participants were healthy, had no prior sexual activity and did not have prior exposure to HPV or any HPV vaccine or vaccine containing AS04. The primary objective of the study was to investigate molecular mechanisms of cross-neutralizing properties of Cervarix^®^ compared to Gardasil-4^®^. Gardasil-4 was used, as Gardasil-9 had not yet been introduced at the time of the study. All study procedures adhered to ICH and GCP guidelines. The study was approved by the Ethics Committee of Ghent University Hospital and by the Belgian Federal Agency for Medicines and Health Products (FAHMP)(EudraCT: 2013-002340-90, NCT 01914367). Informed consent was obtained from all participants and from both parents.

### Study vaccines

Each sister per twin pair was randomized to receive either Cervarix or Gardasil-4. Both vaccines are recombinant vaccines consisting of virus-like particles (VLP) containing L1 proteins of HPV. Cervarix targets HPV types 16 and 18 and uses Adjuvant System 04 (AS04, Al(OH)3 + TLR4 agonist monophosphoryl Lipid A (MPL)). Each formulation contains 20μg L1 VLP for each antigen, 50μg MPL, and 0.5mg Al(OH)3. Gardasil-4 targets HPV types 6, 11, 16 and 18 and is formulated with amorphous AlHO9PS-3 adjuvant. Each formulation contains 20μg L1 VLP for HPV16 and HPV18, 40μg L1 VLP for HPV6 and HPV11, and 225μg aluminum. For both vaccines, two doses of 0.6mL each, were administered intramuscularly to all participants, 6 months apart.

### Sample collection

Ten mL of blood were collected by venous puncture in serum separation blood collection tubes (Becton Dickinson Vacutainer tubes) from all participants at baseline (pre-dose 1, day 0) and 7 days after the second dose (day 187). Serum was seperated after centrifugation for 10 minutes at 1300-2000g and stored in 500 μL aliquots at -20°C for the determination of neutralizing antibody titers and cytokines. At day 187, 60 mL of blood was collected in lithium-heparin coated tubes (Becton Dickinson Vacutainer tubes) for the isolation of peripheral blood mononuclear cells (PBMCs). PBMCs were not collected at baseline (day 0) because cellular immunity was considered to be completely absent in this naïve population. After 1:2 dilution in Hanks buffered salt solution (HBSS), PBMCs were isolated by density gradient centrifugation (Lymphoprep™), washed twice in HBSS, suspended in freezing solution (10% dimethyl sulfoxide/90% fetal bovine serum v/v), frozen at a concentration of up to 10 million cells/mL and stored in liquid nitrogen.

### Pseudovirion-based neutralization assay

Neutralizing antibodies against HPV types 6, 16, 18, 31, 33, 45, 52 and 58 were determined at day 0 and day 187 by pseudovirion-based neutralization assay (PBNA) as described previously (22). Briefly, pseudovirions were produced in HEK293TT cells and purified by ultracentrifugation in an Optiprep gradient. Pseudovirions comprise HPV L1 and L2 proteins that encapsidate a Gaussia luciferase reporter plasmid. Expression of Gaussia luciferase is quantified by luminescent reaction with the luciferase substrate coelenterazine after transduction of the plasmid into HeLaT reporter cells by pseudovirion infection. The pseudovirion infection is blocked and the the transduction of reporter genes is reduced in the presence of neutralizing antibodies. Serum samples were serially diluted in 3.33-fold increments to achieve a final dilution of 1:40 to 1:180 000 on the neutralization assay. Antibody titers were calculated as serum dilutions inhibiting 50% of the luciferase activity (EC50 value). EC50 values greater than 40 were defined as neutralizing antibody-positive. Sufficiently active HPV11 pseudovirions were not available for this study.

### Cytokine assay

The Meso Scale Multi-Array Technology (Meso Scale Discovery) was used for measurement of cytokine levels. A cytokine panel containing the following analytes was screened: IL2, IL4, IL9, IL10, IL17A, TNFα, IFNα2a, IFNβ, IFNy, TGFβ1, TGFβ2, TGFβ3, using 25 μl of each serum from each donor in duplicates. Samples were randomized to avoid batch effects. Results were extrapolated from the standard curve from each specific analyte and plotted in picograms per milliliter, using the DISCOVERY WORKBENCH v.4.0 software (Meso Scale Discovery).

### Single cell RNA and ATAC sequencing

Single cell multiome (RNA+ATAC) sequencing (Chromium Next GEM Single Cell Multiome ATAC + Gene Expression, Document Number GC000338 Rev F, 10X Genomics) was done using PBMCs collected at day 7. Nuclei were prepared from thawed PBMCs according to 10X Genomics demonstrated protocol (Nuclei Isolation for Single Cell Multiome ATAC + Gene Expression Sequencing, Document Number CG000365 Rev C, 10x Genomics). Frozen cells were thawed and incubated with DNase. Thawed cells were counted, and the viability was determined by staining the cells with Trypan blue. Cell suspensions were lysed to obtain isolated nuclei, according to the manufacturer’s instructions. Briefly, cells are incubated with lysis buffer on ice for 4 minutes, in alignment with previous optimization. Nuclei were washed, resuspended, and the percentage of dead cells was determined by incubating the nuclei with trypan blue and counting using an automated cell counter. Nuclei morphology was determined by staining the nuclei with Hoechst, and nuclei are classified as A – D (A, smooth, uniformly round nuclei with well-resolved edges; B, mostly intact nuclei with minor evidence of blebbing; C, nuclei with ruffled edges; D, nuclei no longer intact.). Only samples with the majority of nuclei type A and absence of nuclei type D are used in the subsequent steps of the protocol. Single Cell Multiome ATAC and Gene Expression (GEX) libraries were prepared using the Chromium Single Cell Multiome ATAC + Gene Expression platform (10X Genomics, Pleasanton, CA). 10,000 nuclei were targeted for each sample. Isolated nuclei were transposed and partitioned into Gel Beads-in-emulsion (GEMs) using the 10x Chromium Controller and Next GEM Chip J. GEMs were visually inspected, and only samples with an opaque and uniform aspect were used for library preparation. ATAC and GEX libraries were generated from the same pool of pre-amplified transposed DNA/cDNA. Representative traces and quantitation of both libraries were determined using Bioanalyzer High Sensitivity DNA Analysis (Agilent, Santa Clara, CA). Sequencing was done on Illumina NovaSeq S4 targeting 20,000 reads per nucleus for gene expression and 25,000 reads per nucleus ATAC-seq.

Fastq files were processed 10x Genomics Cell Ranger v5.0.1 using 10x Genomics Cloud Analysis (23, 24). Reads were mapped to the GRCh38 human reference genome and counted without depth normalization. The filtered count matrix was then analyzed using the Seurat (v5.1.0) (25) and Signac packages (v1.14.0) (26) in R. Low quality cells were identified and removed based on the following criteria: more than 100 RNA unique molecular identifiers, less than 25% mitochondrial read fraction, transcription start site (TSS) enrichment score of more than 2 and more than 200 ATAC fragments in peaks. Doublet cells were identified and removed using the DoubletFinder package in R (27). Peak calling was performed using the CallPeaks function. Data normalization, dimensional reductio and batch correction using Harmony integration was done independently on RNA and ATAC assays, which were then integrated using weighted nearest neighbors method (WNN). Cells were clustered using the Louvain algorithm using the integrated Uniform Manifold Approximation and Projection (UMAP), and global differences between clusters were assessed using Principal component analysis (PCA).Cell annotation was carried out using the reference expression dataset derived from Azimuth (28, 29). Differential Gene Expression per cell type between the two vaccine conditions was analyzed using the FindMarkers function (test.use = ‘wilcox’) in the Seurat package and visualized on volcano plots. The obtained sets of DEGs were also used for hierarchical k-means clustering and z-scaled averaged gene expression per subject for each cell type separately were visualized in heatmaps using pheatmap. Ranked DEGs per cell type were subjected to Gene Set Enrichment analysis (GSEA) using the clusterProfiler package in R (30). Reactome pathways were used (31). Average gene expression of significant pathways was calculated using AddModulescore and were correlated to neutralizing antibody titers. Cell-Cell communication analyses were done using the R packages CellChat (32) and NicheNet (33). A per-cell motif binding site activity score is calculated using chromVAR, utilizing a collection of 746 transcription factors from the JASPAR database. To assess chromatin accessibility changes between the two vaccine conditions, differentially accessible regions (DARs) were identified using the FindMarkers function with the LR test for ATAC data in Signac. Regions with significant differences in accessibility were visualized using heatmaps to highlight vaccine-specific regulatory elements. Motif enrichment analysis was performed on DARs using FindMotifs, identifying overrepresented transcription factor binding motifs that may play a role in vaccine-induced immune responses. The top enriched motifs were visualized using MotifPlot, displaying sequence logos that represent nucleotide conservation at these regulatory elements. To further explore transcriptional regulation, CoveragePlot was used to integrate chromatin accessibility and gene expression at key loci. ATAC-seq peaks at this locus suggested potential regulatory elements and links between regulatory regions and gene promoters were overlaid to infer chromatin interactions.

### Statistical analysis

Geometric means of neutralizing antibody titers were calculated for each vaccine at each timepoint. Log10 transformed titers are presented in boxplots (median and IQR). The Mann-Whitney U test was used to evaluate differences in neutralizing antibody titers at day 187. To assess the overall difference in neutralizing antibody titers of all HPV types between twin sisters, the Euclidian distance was calculated as follows:


$$EuclidianDistance=sqrt\left(sum\left({\left(a-b\right)}^{2}\right)\right)$$

Where a and b are the neutralzing antibody titers for each HPV type. HPV6 was not included in this calculation due to an anticipated increase in Euclidian distance, stemming from significantly higher neutralizing antibody titers following Gardasil-4 (which includes HPV6 antigen) compared to Cervarix (which does not contain HPV6). Cytokine concentrations were log10-transformed and tested for significant differences using the Kruskal-Walis test. Pearson correlation coefficients were calculated to evaluate associations between cytokine levels and HPV-type specific neutralizing antibody titers. Differences between the vaccines in relative frequencies of different cell types were analyzed using the Wilcoxon rank-sum test with Bonferoni correction for multiple comparisons. Differentially expressed genes were identified using the Wilcoxon rank-sum test and p-values were adjusted for multiple testing using the Benjamini-Hochberg false-discvery rate (FDR) procedure. Statistical significance was defined as an adjusted p-value ≤ 0.05 and a log2-fold-change > 1. Correlation analyses between module scores of genes in enriched pathways and HPV neutralizing antibody titers were conducted using Kendall’s rank correlation coefficients using the cor.test function in R. Hierarchical clustering was performed on the correlation matrix to group similar rows based on pairwise distances. The Euclidean distance was computed using the dist function, and the hclust function with the complete linkage method was applied for clustering. Continuous data are presented as mean (±SD) or median (IQR), while categorical data are shown as N (%). P-values< 0.05 were considered statistically significant. All analyses were done using R and R Studio.

## Results

### AS04 enhances neutralizing antibody responses

**Figure 1:**
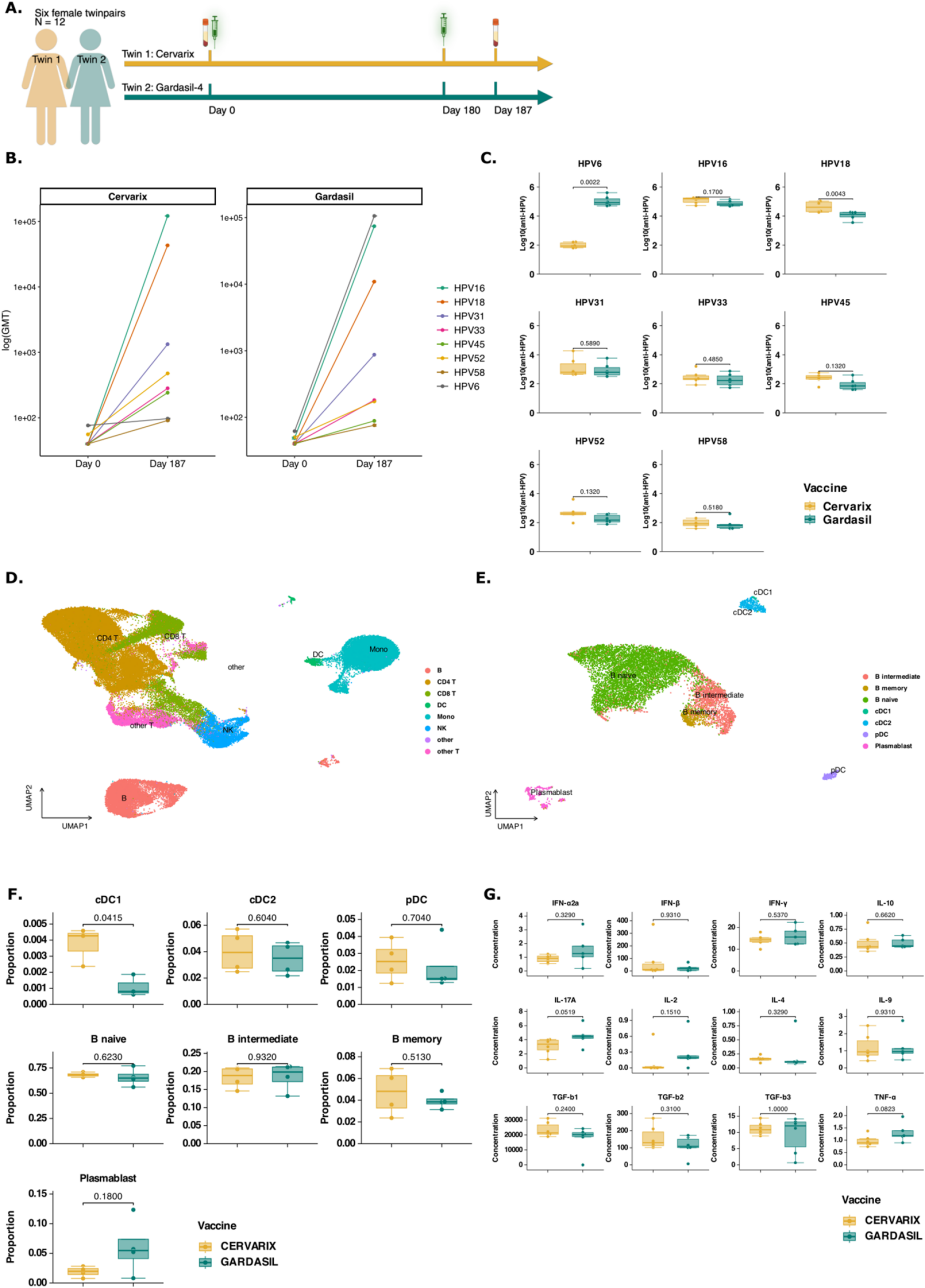
Cervarix induces higher neutralizing antibody titers against closely related HPV types and enhances DC and Memory cell frequencies in peripheral blood. **(A)** Study design: Six female homozygotic twins (n = 12) were vaccinated with either Cervarix, containing AS04, or Gardasil-4, containing alum. All participants were HPV-naive and received the vaccine at the recommended age of 9-13 years in Belgium. Each regimen consisted of two doses administered six months apart (day 0 and day 180). Serum samples were collected on day 0 (pre-vaccination) and seven days after the second dose (day 187), while blood for PBMC isolation was collected on day 187 only. **(B)** Neutralizing antibody titers: neutralizing antibody titers against HPV6, 16, 18, 31, 33, 45, 52 and 58 were measured using a pseudovirion-based neutralizing assay in all participants at both timepoints. Geometric mean titers (GMT) were calculated for each HPV type and vaccine. The line graph presents log-transformed GMT on day 0 and day 187 for each vaccine separately, with colored line representing specific HPV types. **(C)** Neutralizing antibody levels on day 187: Boxplots display the median (IQR) log-transformed neutralizing antibody titers on day 187 for each HPV type, grouped by vaccine (orange: Cervarix, green: Gardasil-4). **(D)** Single cell RNA sequencing analysis: A total of 79,817 cells were analyzed using single-cell RNA sequencing. The UMAP plot visualizes all single cells after dimensionality reduction, clustering, and annotation. **(E)** A subset of all DCs and B cells were selected for further investigation. The UMAP plot visualizes the subset of single cells after dimensionality reduction and re-clustering. **(F)** Cell frequencies: Boxplots show the median (IQR) of relative cell frequencies per vaccine. **(G)** Cytokine concentrations: Boxplots show median (IQR) for each measured cytokine. Mono, monocytes; NK, Natural Killer cells; cDC1/cDC2, conventional dendritic cells type 1 or type 2; pDC, plasmacytoid dendritic cell; CD4, CD4+ T cells; CD8, CD8+ T cells; GMT, geometric mean titer; HPV, human papilloma virus; significance was assessed by the actual p-values by Wilcoxon rank test.

To investigate the effect of AS04 on antibody responses and its molecular mechanisms, six female homozygotic twins (n = 12), were vaccinated with either Cervarix, containing AS04, or Gardasil-4, containing alum. All participants were naive for HPV and received the vaccine at the recommended age of 9-13 years in Belgium. Each regimen consisted of 2 doses, 6 months apart (day 0 and day 180) (**Figure 1A**). Serum was collected at day 0 and 7 days post-second dose (day 187), while blood for peripheral blood mononuclear cell (PBMC) isolation was collected at day 187 only. The neutralizing antibody response was measured using a pseudovirion-based neutralization assay (22). Both vaccines target the L1 protein of HPV16 and 18 and successfully induced neutralizing antibodies (**Figure 1B**), with a marked increase observed from day 0 to day 187 (**Supplementary Table 1**). Gardasil-4 additionally targets HPV6 and 11 (type 11 not measured). Neutralizing antibodies against HPV31, 33, 52 and 58 (closely related to HPV16) and HPV45 (closely related to HPV 18) were also increased by day 187 (**Figure 1B**). On day 187, neutralizing antibody titers against HPV18 were significantly higher in participants vaccinated with Cervarix, consistent with previous studies demonstrating Cervarix’s superior response to HPV18 compared to Gardasil-4 (**Figure 1C**; **Supplementary Table 1**). Additionally, a trend (p<0.2) towards higher neutralizing antibody titres against HPV45 and HPV52 after Cervarix vaccination was observed. Conversely, titers against HPV6 were significantly higher following Gardasil-4 vaccination, as this antigen is included in Gardasil-4 but not in Cervarix (**Figure 1C**). While titers against HPV16 and other related types (31, 33, and 58) showed no significant differences between vaccines, a limited trend favoring Cervarix was observed (**Figure 1C**). Overall, these findings confirm that Cervarix has a greater capacity to enhance cross-protective antibody responses compared to Gardasil-4, thereby broadening the immune response. On day 187, no differences in serum cytokine concentrations were observed between vaccines, likely reflecting the expected return of the inflammatory response to baseline by this time (**Figure 1G**; **Supplementary Table 1**).

### AS04 is associated with increased frequencies of dendritic cells

Next, we investigated the differential effect of AS04 on transcriptional and epigenetic changes in PBMCs. Four twins (n = 8) representative of the biggest differences in neutralizing antibody titers across all types of HPV, except HPV6, were selected by calculating the Euclidian distance (**Supplementary Figure 1A**). Nuclei from these eight PBMC samples collected on day 187 were subjected to multi-ome single cell RNA and ATAC sequencing (10X Genomics). In total, 79,817 cells were sequenced, and 71,767 high-quality cells were retained for analysis, including clustering and annotation using the Azimuth PBMC reference (**Figure 1D**; **Supplementary Figures 1B–M**). Relative frequencies of all major cell types are shown in **Supplementary Figure 2A** (**Supplementary Table 2**). AS04 has previously been shown to activate DCs, resulting in better antigen presentation, rather than directly activating CD4+ T cells (20). We selected all cells annotated as DC, monocyte or B cell thorugh automated annotation using Azimuth and subsequently reclustered them (**Supplementary Figure 2B**). The annotation of each cell type was verified by plotting the average expression of common dendritic cell markers (**Supplementary Figure 2C**) or monocyte markers (**Supplementary Figure 2D**). We observed that the DC population was well defined and distinctly clustered apart from the monocytes. In contrast, the monocyte population was more heterogenous, which could potentially impact the accuracy of the results. Therefore, we focused on DCs and B cells to investigate mechanisms underlying cross-protective humoral immune responses. Finally, all annotated DCs and B cells were selected and reclustered (**Figure 1E**). A statistically significant difference in relative frequencies of conventional DCs type 1 (cDC1) was found using Wilcoxon Rank-sum test (**Figure 1F**). Cervarix showed a limited trend towards higher proportions of cDC2 and plasmacytoid dendritic cells (pDC). Although not statistically significant, a higher proportion of memory B cells was observed following Cervarix vaccination, but a decreased proportion of plasmablasts compared to Gardasil-4 (**Figure 1F**; **Supplementary Table 2**).

### AS04 enhances NOTCH signaling through gene expression in cDC1, stimulating cell cycling in memory B cells, which correlates with breadth of neutralizing antibodies

To further investigate the function of immune cells, differentially expressed genes (DEG) in each cell type were identified and analyzed using gene set enrichment analysis (GSEA) (**Supplementary Table 3**). Ribosomal genes were among the most differentially expressed genes and genes related to RNA translation, cell metabolism and cell proliferation pathways were enriched in the Cervarix group, which indicates an enhanced cellular machinery for protein synthesis and can support a more robust immune response by facilitating the activation and proliferation of immune cells.

**Figure 2:**
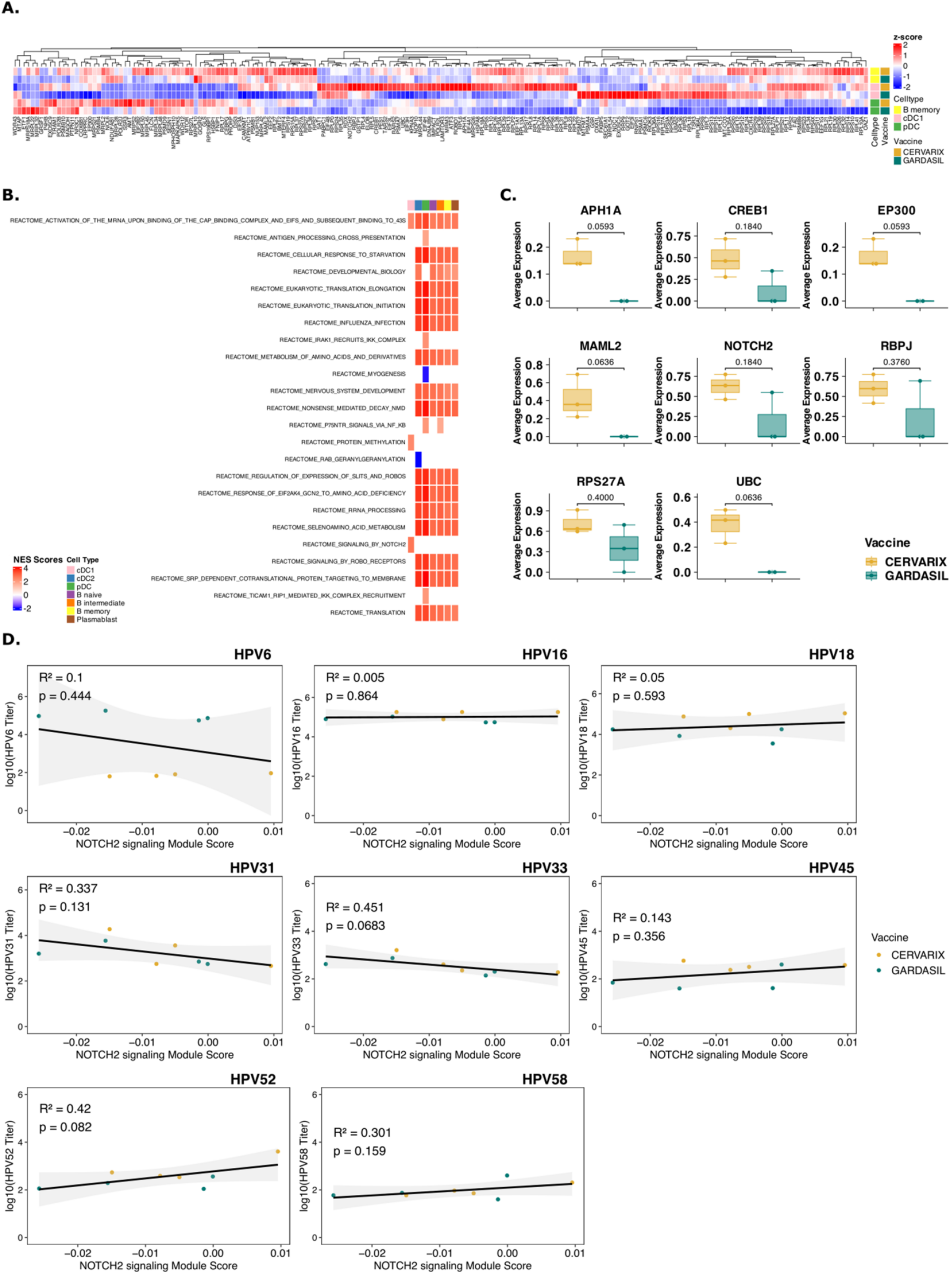
Cervarix induces transcriptional responses in DCs and memory B cells that correlate with antibody titers. **(A)** Clustered heatmap of all genes involved in identified enriched pathways in cDC1, pDC, and B memory cells. Color represents column-wise z-scores. **(B)** Heatmap of normalized enriched scores (NES) for significantly enriched reactome pathways in cDC1, pDC, and memory B cells. All pathways that were enriched in any of DC subsets are shown. Additional pathways enriched in B cell subsets are included in **Supplementary Table 3**. **(C)** Boxplot showing the median (IQR) of average gene expression of NOTCH2 signaling pathway genes in cDC1, grouped by vaccine. **(D)** Scatter plots showing correlation between the module score of NOTCH2 signaling pathway per participant with neutralizing antibody titers against all measured HPV types. cDC1, conventional dendritic cells type 1; cDC2, conventional dendritic cells type 2; pDC, plasmacytoid dendritic cell; NES, normalized enrichment score.

In cDC1 and pDCs, there was increased expression of genes involved in RNA translation, protein folding and cell-cycling, -development, and -apoptosis (**Supplementary Figures 2E–F**). In memory B cells ribosomal genes were also the most overexpressed genes in B cells following Cervarix, compared to Gardasil-4 (**Supplementary Figure 2G**). Overall, cervarix increased the expression of genes involved in RNA translation, cell metabolism and cell proliferation pathways in DCs and B cells (**Figure 2A**). DCs showed enrichment of cell recruitment and cell signaling pathways, while memory B cells showed enrichment of cell survival and proliferation pathways (**Figures 2A, B**). Interestingly, NOTCH2 signaling, important in cell differantion and an important regulator of Th2 immunity, was one of the three enriched pathways identified in cDC1, and possibly involved in the observed increase in cell proliferation (**Figures 2A, B**). Indeed, genes and transcription factors involved in the NOTCH2 signaling, including NOTCH2, MAML2, and RBPJ (**Figures 2A–C**) showed increased average gene expression in subjects receiving Cervarix.

Moreover, transcription factors and genes involved in cell cycling, cell survival and anti-apoptosis such as CDK7, CYC1, MICA, and OPA1, were among the top expressed genes in memory B cells (**Supplementary Figure 2G**). Indeed, mRNA translation, protein folding, and cell cycling were pathways significantly enriched after Cervarix across cell types.

We next sought to determine whether the identified pathways in cDC1, pDC, and B memory cells correlated with higher neutralizing antibody titers, independent of the vaccine. First, Module scores, which is the average expression level of all genes of the respective pathway, for all significantly enriched pathways were calculated. Then, Kendall’s rank correlation coefficients were calculated between the module score and neutralizing antibody titers for various HPV types (**Supplementary Table 4**). Hierarchical clustering based on pairwise distances was performed, and the resulting dendrogram was used to order rows in the heatmap (**Supplementary Figure 2H**). Clustering revealed the two groups of HPV types: HPV18-45-52-58, and HPV31-33 based on correlation coefficients, which mostly correspond to the A7 (HPV18-45) and A9 (HPV16-31-33-52-58) HPV groups. HPV6 and HPV16 clustered seperately. The pathways upregulated after Cervarix vaccination, primarily those related to cell cycle and RNA translation, positively correlated with neutralizing antibody titers for HPV18-45-52-58, aligning with the higher neutralizing antibody titers observed for these types after Cervarix. Ribosomal gene expression in all cells and Slit-Robo signaling in pDCs and memory B cells, also showed weaker positive correlations with neutralizing antibody titers for other HPV types. Conversely, pathways primarily associated with immune responses, such as B cell receptor activation, were upregulated after Gardasil-4 vaccination and positively correlated with neutralizing antibody titers for HPV6-16-31-33. Most interesting, NOTCH2 signaling, correlated positively with HPV18-45-52-58 and HPV16 (**Figure 2D**).

To further explore differences in gene expression based on the breadth of neutralizing antibody responses, subjects were categorized as “high breadth responders” [n=4 (Cervarix: n=3, Gardasil-4: n =1)] and “low breath responders” [n=4 (Cervarix: n=1, Gardasil-4: n =3)] based on the sum of neutralizing antibody titers across all HPV types, with the median serving as threshold. Differential gene expression analysis (**Supplementary Figures 3A, B**; **Supplementary Table 4**) and GSEA identified similar enriched pathways (**Supplementary Figure 3C**; **Supplementary Table 4**). High breadth responders showed increased ribosomal gene expression and enrichment of pathways related to RNA translation, cell metabolism and development in DCs and B cells.

These findings suggest that Cervarix promotes cell differentiation and maturation via DC signaling, persisting at least until day 7, with a greater reliance on memory cells compared to Gardasil-4. Moreover, NOTCH2 signaling by cDC1 appears to enhance cell proliferation and development of memory B cells. This induced NOTCH2 signaling seems to favor memory B cell lineage commitment over plasmablast differentiation. Consequently, the resulting immune profile supports long-term, cross-neutralizing humoral responses rather than short-lived plasmablast expansion. However, this conclusion is based solely on gene expression data. Memory B cells were not quantified using conventional flow cytometry, and the observed cell cycling and differentiation were not confirmed with established proliferation markers such as Ki-67 or CD69.

### AS04 increases chromatin accessibility of NOTCH-related genes in cDC1s

**Figure 3:**
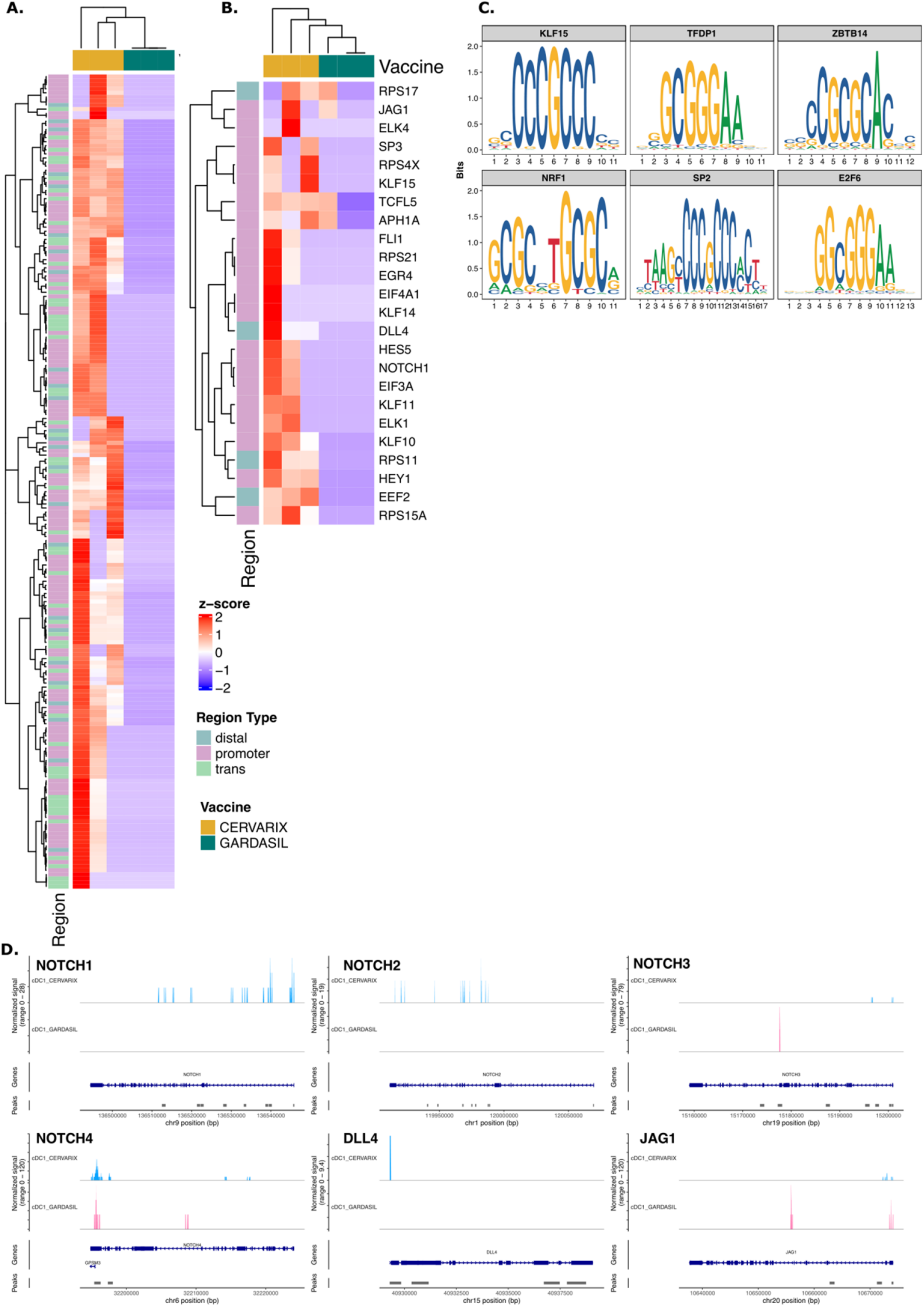
Cervarix enhances chromatin accessibility of NOTCH genes in cDC1. **(A)** Heatmap showing normalized chromatin accessibility at the top 200 DARs in cDC1 for each subject. Regions were classified as follows: promoter −2,000 bp to +500 bp; distal −10 kbp to +10 kbp – promoter; trans< −10 kbp or > +10 kbp. **(B)** Heatmap of normalized accessibility of NOTCH-related DARs in cDC1 for each subject. **(C)** The top enriched motifs identified from NOTCH-related DARs in cDC1. **(D)** Chromatin accessibility at the locus of NOTCH ligands (NOTCH1, NOTCH2, NOTCH3, NOTCH4, DLL4, JAG1) in cDC1 grouped per vaccine. The coverage tracks represent the aggregate signal of transposase-accessible regions, with peaks indicating open chromatin regions. The plot extends 50 bp upstream and 10 kb downstream of the gene to capture potential regulatory elements. cDC1, conventional dendritic cells type 1; cDC2, conventional dendritic cells type 2; pDC, plasmacytoid dendritic cell.

To investigate epigenetic changes in DCs induced by AS04, we performed single cell ATAC sequencing on PBMCs collected on day 7 after the second dose. In cDC1 and pDCs, we analyzed genomic regions with increased chromatin accessibility, which may indicate prior training of these cells. The top 200 differentially accessible regions (DARs) in Cervarix compared to Gardasil-4 are shown in **Figure 3A** for cDC1 and **Supplementary Figure 4A** for pDC (**Supplementary Table 5**). Interestingly, several NOTCH-related genes exhibited increased accessibility after Cervarix (**Figure 3B**). Thes genes included promotor regions of EGR4, HES5, HEY1, and NOTCH1, as well as distal regions of DLL4 and EEG2. The top six enriched motifs in these NOTCH-related DARs (KLF15, TFDP1, ZBTB14, NRF1, SP2, and E2F6) (**Figure 3C**) correspond to transcription factors involved in cell recruitment, gene regulation, cell differentiation and mitochondrial biogenesis. While DARs associated with NOTCH genes were identified in pDCs, the differences were less pronounced (**Supplementary Figure 4B**). The top six enriched motifs in NOTCH-related DARs in pDCs (KLF15, EGR1, SP14, ZBTB14, NRF1, and EFR2) were also implicated in cell recruitment, gene regulation and cell differentiation (**Supplementary Figure 4E**) (34–37). Indeed, the NOTCH2 locus showed more open chromatin in cDC1 after Cervarix vaccination (**Figure 3D**), and chromatin accessibility of NOTCH-related ligands (NOTCH1, DLL4, JAG1) in cDC and pDC clusters, and of NOTCH-related target genes (HEY1, HES5, ELK1) in the B cell cluster (**Figure 3D**). While loci of NOTCH-related ligands (NOTCH1, NOTCH2, NOTCH3, NOTCH4, DLL4, and JAG1) showed more peaks indicating more open chromatin after Cervarix, this was more pronounced in conventional than plasmacytoid DCs (**Figure 3D**; **Supplementary Figure 4F**). The enhanced chromatin accessibility, primarily in promotor regions of NOTCH genes in DCs, aligns with increased transcription and may suggest prior training of these cells by the first dose, although this cannot be definitively confirmed.

### AS04 enhances signaling from DCs to adaptive immune cells and drives cell survival

**Figure 4:**
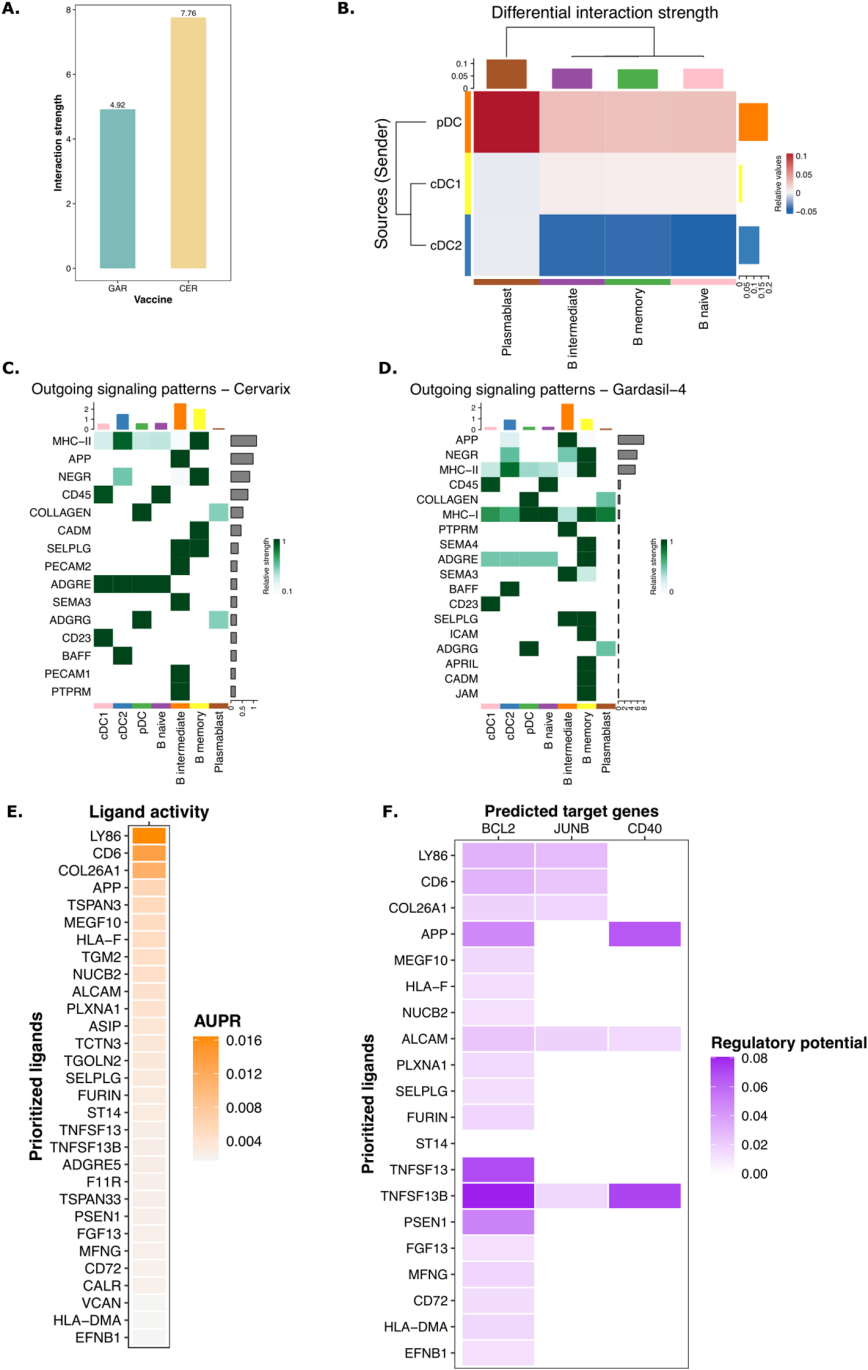
Enhanced signaling by DCs via NOTCH after Cervarix vaccination. **(A)** Bar plot showing the total strength of interactions per vaccine. **(B)** Heatmap comparing the differential strength of interactions between Cervarix and Gardasil-4. Rows represent sending cells (y-axis), while columns indicate receiving cells (x-axis). Red denotes stronger signaling in Cervarix, while blue indicates stronger signaling in Gardasil-4. The heatmap is clustered based on the differential strength of interactions. Bar plots along the rows and columns indicate the total strength of signals sent or received, respectively.C-D. Heatmaps showing outgoing signals for each cell type in Cervarix **(C)** and Gardasil-4 **(D)**. “Outgoing” refers to signals sent by the specified cell types. Signals are ranked by ‘importance’ and the extent to which cells utilize them. Bar plots on the columns represent the total number of signals sent by each cell type, while the bar plots on the rows indicate the total number of signals sent overall. Green highlights the relative strength of the outgoing signal. E-F NicheNet analysis of DC signaling to memory B cells. **(E)** Ligand activity plot highlighting the most important ligands sent by cDC1 and pDC to memory B cells. **(F)** Target gene heatmap showing the influence on expression of BCL2, JUNB and CD40 in memory B cells (columns) by the top ligands sent by DCs (rows). cDC1/cDC2, conventional dendritic cells type 1 or type 2; pDC, plasmacytoid dendritic cell; AUPR, area under the precision recall curve.

We next sought to examine in detail the molecular signals mediating communication from DCs to adaptive immune cells. Using the CellChat package (32), we analyzed cell-cell interactions, defined as ligand-receptor interactions inferred from the gene expression matrix and compared to a literature validated database of ligand-receptor interactions, between DCs and B cells after Cervarix and Gardasil-4 vaccination. A comparison of interaction strength, as a measure of communication quality, between DCs and B cells cells revealed stronger interactions following Cervarix vaccination (**Figure 4A**). Specifically, interactions between cDC1/pDC and B cells were stronger after Cervarix, whereas interactions between cDC2 and B cells predominated after Gardasil-4 (**Figure 4B**). Stronger interactions to memory B cells occurred after Cervarix vaccination.

For both vaccines, pDCs were identified as the primary ligand-sending cells, with MHC-I and MHC-II being the most frequently used ligands (**Figures 4C, D**). However, after Cervarix vaccination, cDC1 and cDC2 played a more prominent role in sending signals. These signals included ligands that are important in recruitment, adhesion, and activation of cells (SELPLG, PECAM1, ADGRG, BAFF). Interestingly, several of these ligands, BAFF, CD86, and PECAM1, strongly impact B cell survival. Additionally, MHC-II presented by cDC1 and cDC2 was more important after Cervarix vaccination compared to Gardasil-4 (**Figures 4C, D**).

To further investigate the functional outcomes of these interactions, we used NicheNet to identify genes differentially expressed after Cervarix vaccination in recipient cells (memory B cells) upon receiving these signals from DCs. Similarly, signaling from DCs to memory B cells was evidenced by the activity of several ligands involved in recruitment and adhesion (SELPLG, ADGRE5, APP) of B cells and allowing for stable synapses needed for effective communication.

Again, ligands, including PSEN1, MFNG (NOTCH signaling), CD6 (co-stimulation), BAFF, and APRIL (B cell activating factor), that stimulate cell proliferation and survival signals in memory B cells were found, (**Figure 4E**). These ligands further enhanced memory B cell survival by inducing the expression of survival-associated genes like BCL2, CD40 and JUNB (**Figure 4F**; **Supplementary Figure 5**). Although we did not quantify these ligands at the protein level, overall, the gene expression data overall suggest that, in addition to increased NOTCH signaling by DCs, these cells facilitate the recruitment, proliferation, and survival of B memory cells, thereby enhancing the adaptive immune response.

### Cervarix enhances NOTCH signaling by T helper cells

**Figure 5:**
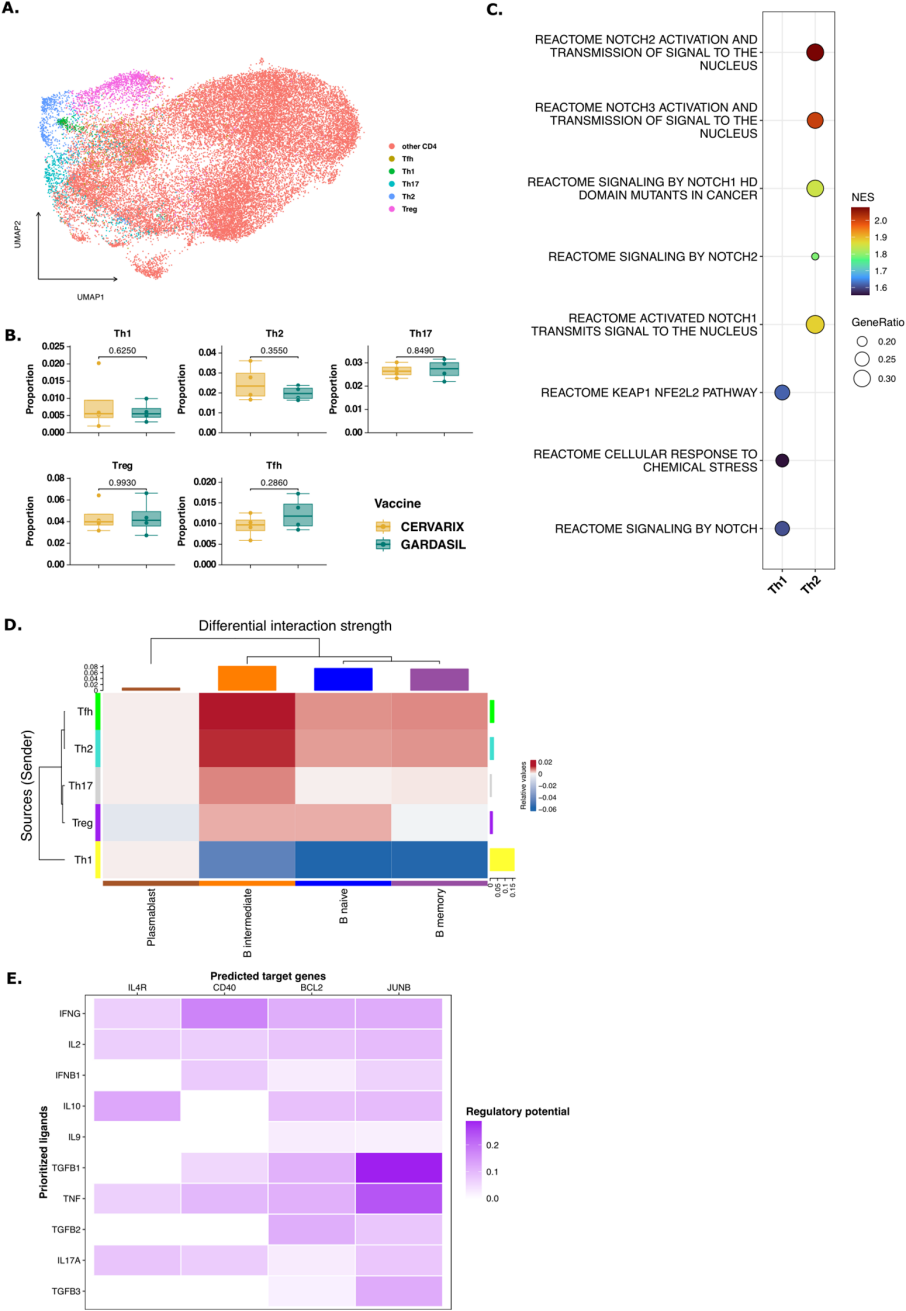
T help via NOTCH signaling to B memory cells. **(A)** UMAP projecting displaying all CD4+ T cells after dimensionality reduction and clustering and manual annotation of T helper subsets. **(B)** Boxplots representing the median (IQR) relative frequencies of CD4+ T cell subsets, stratified by vaccine (orange: Cervarix, green: Gardasil-4). **(C)** Dotplot showing significantly enriched pathways related to NOTCH signaling in Th1 and Th2 cells. Positive NES indicates a pathway enriched in Cervarix. **(D)** Heatmap showing the differential strength of interactions between cell types. Rows represent sender cells (y-axis), and columns represent receiver cells (x-axis). Blue indicates stronger signaling in Cervarix group, and red indicates stronger signaling in Gardasil-4 group. The heatmap is clustered by the differential interaction strength, with bar plots on the rows and columns indicating the weight of signals sent or received, respectively. **(E)** NicheNet analysis of Th2 signaling to memory B cells. Target gene heatmap showing the expression of genes in memory B cells (columns) influenced by several cytokines sent by Th2 cells (rows). **(F)** Boxplots showing the median (IQR) of average expression of Immunoglobulin-related genes in B memory cells, stratified by vaccine. Tfh, follicular T helper cell; Th, helper T cell; Treg, regulatory T cell.

Since the NOTCH signaling pathway appears to play an important role, we investigated whether this also leads to the activation of Th2 cells, as NOTCH signaling is a hallmark feature of these cells. To investigate whether Cervarix impacts T helper cells, potentially facilitating broader help and extended affinity maturation, we re-clustered all CD4+ T cells (**Figure 5A**). For each cluster, the average expression of common T cell markers was plotted and clusters were manually annotated as Th1, Th2, Th17, follicular helper T cells (Tfh), regulatory T cells (Treg), or other CD4+ T cells. No notable difference in T helper cell frequencies were observed (**Figure 5B**; **Supplementary Table 6**), but several NOTCH pathways were significantly enriched in Th2 andTh1 cells, after Cervarix vaccination, confirming the involvement of NOTCH signaling in Th2 and Th1 functionality (**Figure 5C**).

We next examined which helper T cells provided enhanced help to B memory cells after Cervarix vaccination. Differences in communication between Th cells and B cells between the two vaccines were again assessed using the Cellchat package. This analysis revealed stronger interactions between all Th cells, except Th1, and B memory, B intermediate and B naive cells after Cervarix compared to Gardasil-4 (**Figure 5D**). Conversely, Th1 signaling was proportionally stronger after Gardasil-4 (**Figure 5D**). Using NicheNet, we analyzed next which cytokines were being sent by Th cells to induce target gene expression genes in memory B cells. Indeed, a broad set of genes relating to several CD4+ T helper subsets, were identified as ligands. These cytokines were found to stimulate the expression of genes that were upregulated in B memory cells following Cervarix vaccination, including BCL2, JUNB and CD40 (**Figure 5E**). Additonally, ligands other than cytokines - such as Nothc-related genes like ADAM12 (involved in NOTCH cleavage) and CD6 (a co-stimulatory molecule) - were sent by Th cells, highlighting the role of NOTCH signaling in T cell help (**Supplementary Figure 5**). Overall, these findings suggest that Cervarix also induces NOTCH signaling in Th cells, which may contribute to the enhanced activation of memory B cell.

### NOTCH signaling, cell cycling and survival gene signatures are associated with antibody breadth in other vaccines

**Figure 6:**
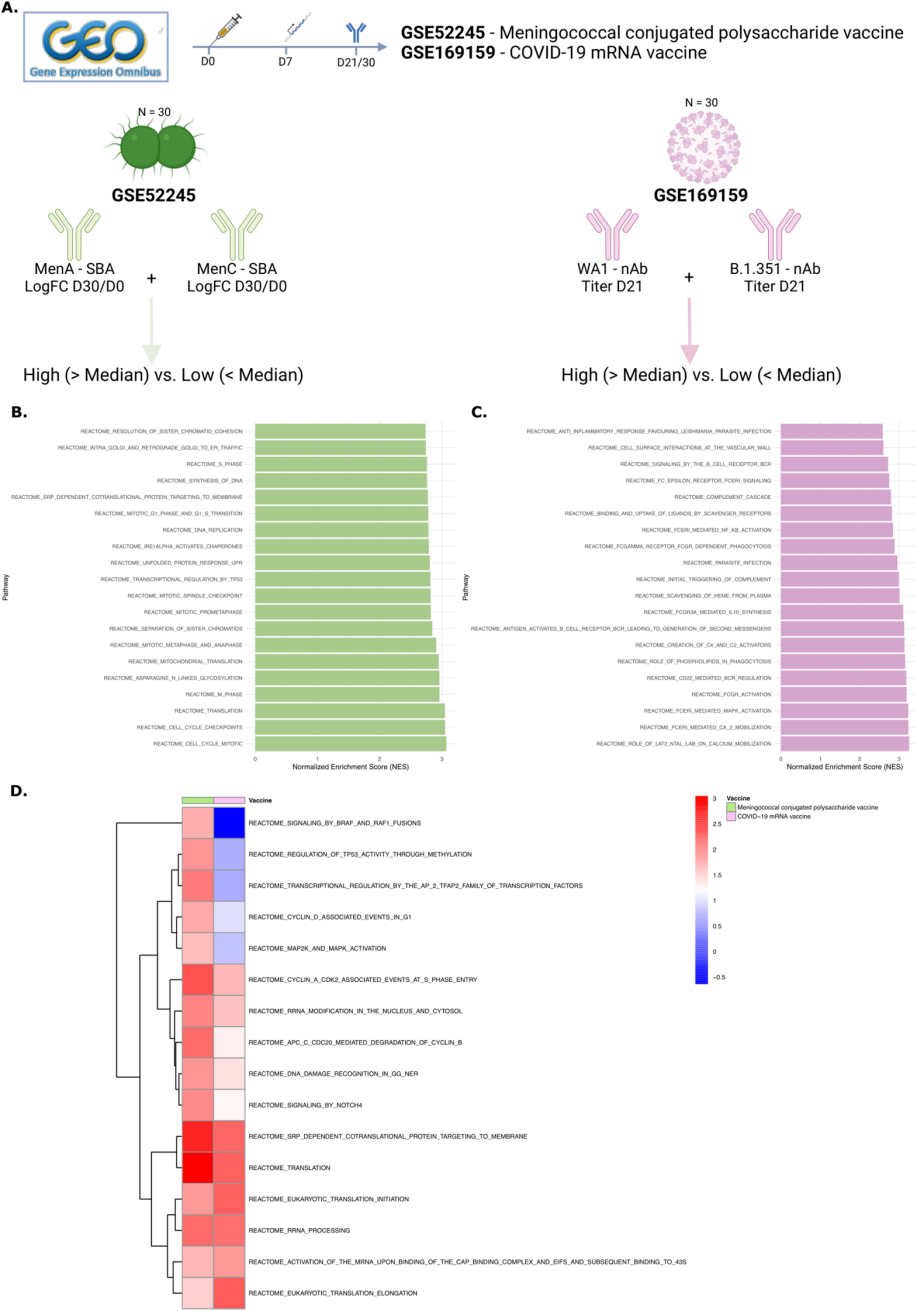
NOTCH signaling and cell cycling and survival gene signatures are associated with antibody breadth in other vaccines. **(A)** The Gene Expression Omnibus (GEO) database was searched for publicly available gene expression data that included antibody titers across multiple strains or pathogen types. Two relevant studies were identified. The first examined a conjugated polysaccharide vaccine against meningococcus, with serum bactericidal antibody titers available for MenA and MenC. The second study analyzed gene signatures of an mRNA COVID-19 vaccine and their relationship with neutralization antibody titers against two SARS-CoV-2 strains. For both studies, titers across strains were summed, and subjects were categorized into high and low breadth groups based on the median value. **(B-C)**. Bar charts displaying the top 20 enriched pathways in high- vs. low-breadth subjects for GSE52245 **(B)** and GSE169159 **(C)**. **(D)** Heatmap showing the NES of pathways commonly enriched in both vaccines, clustered per vaccine. NES, normalized enrichment score; GEO, gene expression omnibus; nAB, neutralizing antibodies; SBA, serum bactericidal assay.

Lastly, we aimed to investigate whether the gene signature identified here, including NOTCH signaling and increased cell survival, could be associated with breadth of the antibody response to other vaccines. The ImmuneSpace database was searched for studies investigating vaccine-induced immune responses that included both gene expression (RNA sequencing/microarray) and antibody titers (ELISA, microneutralization, or functional assays). The Gene Expression Omnibus (GEO) database was searched for publicly available gene expression data that also included antibody titers across multiple strains or types of the pathogen. Two relevant studies were identified, data on antibody titer measurements were not available for the other studies. (**Figure 6A**). The first study assessed a conjugated polysaccharide vaccine against meningococcus, with serum bactericidal antibody titers available for MenA and MenC (38). The second study examined the gene signatures of an mRNA COVID-19 vaccine and its relationship with neutralization antibody titers against two SARS-CoV-2 strains (39). For both studies, titers across strains were summed, and subjects were classified into high and low breadth groups based on the median value. Indeed, the top 20 significantly enriched pathways in high-breadth versus low-breadth responders after meningococcal vaccination included cell cycling and RNA translation processes (**Figure 6B**; **Supplementary Table 7**). After COVID-19 vaccination, the top 20 significantly enriched pathways included B cell receptor activation and cell migration pathways (**Figure 6C**). Furthermore, almost all the significantly enriched pathways after Cervarix identified in our dataset were also enriched in high-breadth responders for both vaccines (**Figure 6D**). These findings partially validate our results in this small population.

## Discussion

In this study, we analyzed blood samples collected before first vaccination and 7 days after the the second vaccine dose administered to six homozygotic twin sisters. Our aim was to investigate the molecular mechanisms by which Cervarix, containing the adjuvant system AS04, induces a stronger cross-protective antibody response to oncogenic HPV types not included in the vaccine compared to Gardasil-4. Both vaccines generated high neutralizing antibody titers against HPV16 and HPV18. However, Cervarix elicited significantly higher neutralizing antibodies against HPV18 with a trend toward higher titers against HPV31, 33, 45, 52 and 58, compared to Gardasil-4.

At the cellular level, this response was associated with an increased relative frequency of DCs and memory B cells. Functionally, we observed enhanced DC recruitment and NOTCH signaling by cDC1, leading to active cell proliferation and survival of memory B cells, even seven days after the second dose. These findings correlated with higher neutralizing antibody titers and a broader humoral response. This was accompanied by heightened cell-cell communication between Th cells and B memory cells, which stimulated the expression of cell survival genes, potentially facilitating prolonged affinity maturation.

Consistent with previous research, our findings confirm that Cervarix induces higher neutralizing antibody titers against HPV18 and the closely related HPV45 and HPV52 as compared to Gardasil-4 (15, 18). Studies attribute this superior response to the enhanced immunogenicity of the AS04 adjuvant (40, 41). AS04 has been shown to increase both the magnitude and durability of immune responses induced by HPV and hepatitis B vaccines (42). In preclinical models, AS04 was shown to activate APCs through TLR4, without directly targeting CD4+ T cells or B cells (20). Our study corroborates these findings by showing recruitment of cDC1 and pDCs which play a pivotal role in bridging innate and adaptive immunity. The activation of Toll-like receptor 4 (TLR4) by AS04 accounts for these robust DC responses, as TLR4 stimulation is well-recognized for enhancing antigen presentation and subsequent T-cell activation (43).

The upregulation of NOTCH-related genes in cDC1 cells in our dataset further suggests a role for NOTCH signaling in shaping adaptive immunity via antigen-presenting cells. Indeed, it has been shown that NOTCH signaling optimizes cDC1 differentiation *in vitro*, leading to better antigen presentation (44). In mice, NOTCH-ligand expressing DCs were found to better regulate T-cell effector functions leading to reduced tumor growth (45). Moreover, it was shown that NOTCH1 signaling increased antigen responsiveness in CAR-T cells (46). NOTCH signaling has also been shown to be critical for the differentiation of B cells to antibody-secreting cells, as its’ signaling enhances CD40 expression, as was seen in our cell-cell communication analyses, thereby supporting long-term humoral immunity (47). NOTCH signaling is well known to contribute to both T and B cell activation and differentiation (47–49). Thus, the contribution of NOTCH signaling to higher cell survival and proliferation on day 187, could allow extended time for affinity maturation, enhancing the vaccine’s capacity to generate cross-protective responses against related HPV types. This nuanced immune modulation by Cervarix may be a critical factor differentiating its overall immunogenicity profile.

Notably, gene regions associated with NOTCH signaling also exhibited greater accessibility in cDC1, indicating that the first dose of AS04 or prior antigenic encounters may have epigenetically primed these cells, which could contribute to enhanced responsiveness upon vaccination. Although, this aligns with other adjuvants such as AS03, inducing trained immunity (50), we cannot confirm this due to the lack of baseline samples. However, such epigenetic modifications may facilitate a more robust adaptive immune response, potentially explaining the differential vaccine-induced memory B cell activation observed in our study.

While the role of Th1 and pro-inflammatory responses in antiviral immunity is well-established, our findings demonstrate that AS04-driven Th activation and enhanced memory cell recruitment offer a complementary pathway for achieving cross-protection. the robust interaction between Th cells and B cells, highlighted by our cell-cell communication analysis, underscores the critical role of Th-dependent immune pathways. This process may enhance the breadth of neutralizing antibody responses and distinguish Cervarix from Gardasil-4.

Additionally, systems serology analyses have highlighted the importance of qualitative antibody attributes, such as Fc-mediated effector functions, in determining vaccine efficacy (19, 51, 52). Compared to Alum, AS04 has been shown to enhance antibody avidity, Fc-receptor functions, and memory B cell recall, although to a lesser extent than some other adjuvant systems (19, 51). More recently, studies have demonstrated that both Cervarix and Gardasil-4 elicit robust Fc-effector functions. However, Cervarix was found to coordinate these responses more effectively, resulting in higher antibody-dependent complement activating responses (52).

Lastly, the robust immune response against HPV18 observed for both vaccines may have clinical implications, given the association of HPV18 with aggressive cervical cancer phenotypes. Higher titers of cross-neutralizing antibodies, particularly against HPV18-related types, are linked to reduced rates of persistent infection and cervical intraepithelial neoplasia (53).

This study leveraged single-cell RNA sequencing to provide a detailed view of cell-type-specific immune responses. The analysis of transcriptional changes offered insights into the molecular pathways activated by Cervarix. The use of homozygotic twins minimized genetic variability, especially in HLA genes, enabling precise comparisons and enhancing the reliability of observed differences. However, some limitations remain. The twin-based design, while strengthening internal validity, involved a small sample size (n = 12), limiting generalizability of the findings. Indeed, a small number of total cells was analyzed, and while relative proportions are within expected ranges, the absolute number of cells is low. However, the validation in public gene expression data confirmed the correlation of these pathways with breadth of antibody responses. Moreover, several studies found an association of gene signatures related to cell cycling and durable antibody responses (50, 54, 55), including the effort of defining a transcriptional atlas of 13 vaccines (56, 57). Next, immune responses were assessed only seven days after the second vaccination, and baseline cellular immune responses were not measured. While all subjects were HPV naive with no expected pre-existing immunity, a baseline PBMC sample would have allowed identification of trained immunity and of vaccine-specific changes over time. Future longitudinal studies are needed to evaluate antibody persistence and long-term immune memory. Lastly, the primary limitation of this study is that neutralizing antibody titers were the sole functional immunological parameter assessed. Cellular immune responses were not confirmed using conventional flow cytometry. Consequently, the findings reflect overall changes in the PBMC compartment and cannot be directly extrapolated to vaccine- or antigen-specific cellular immune responses. Several approaches to detect antigen-specific B cells were explored, including the use of labeled HPV VLPs. While these methods are relatively straightforward for detecting antigen-specific T cells, they were not feasible for B cells in this study, which were of primary interest. The exclusion of such analyses was mainly due to technical challenges, such as the low frequency of B cells within PBMCs and limited knowledge about the frequency of HPV-specific B cells generated by vaccination. However, the primary focus was on the effect of AS04, particularly on the activation of the innate immune response and innate-to-adaptive communication, processes that are inherently nonspecific. Additionally, quantification of various ligands or gene expression levels using conventional techniques, such as ELISA or qPCR, was not performed. For instance, the detection of HPV-specific IgG subtypes 1 to 4 could have provided further insights into both the cell-mediated (Th1/Th2) and humoral (antibody-dependent cellular cytotoxicity) immunity. Although functional validation was not possible, our conclusions are based on gene exression and chromatin accessibility data, which offer deeper insights into molecular mechanisms of HPV vaccines that warrant further investigations.

In conclusion, AS04 increased DC frequencies, with cDC1 in particular exhibiting enhanced NOTCH signaling, evidenced by increased gene expression and epigenetic modifications likely induced by prior training. This signaling promoted cell survival, RNA translation, and proliferation, particularly in memory B cells up to seven days post-vaccination. Additionally, AS04 expanded T cell help, with the resulting adaptive immune response relying more heavily on memory B cells exhibiting higher survival. While these findings should be further validated through functional immunoassays, the single-cell sequencing data suggest a state of immune activation that could facilitate more effective maturation of adaptive immune cells, possibly explaining the observed increased cross-reactivity with Cervarix. These results emphasize the importance of adjuvant selection in optimizing vaccine efficacy and highlight the potential of AS04 to induce robust, long-lasting immunity.

## Data Availability

The original contributions presented in the study are included in the article/supplementary material, further inquiries can be directed to the corresponding author/s. Gene expression and chromatin accessibility data are available on Gene Expression Omnibus with accession number: GSE299801.

## Supplementary material

Supplementary Figure 1 Single cell RNA and ATAC sequencing quality. **(A)** Euclidian Distances between twin sisters based on neutralizing antibody titers for all HPV types. **(B)** Bargraph showing the number of doublet cells for each subject identified using the DoubletFinder package. **(C-H)** Violin plots showing the distribution of six quality metrics across all cells. Quality metrics were number of reads **(C)**, number of transcripts **(D)**, percentage of mitochondrial genes **(E)**, number of ATAC reads **(F)**, nucleosome signal **(G)**, and TSS enrichments score **(H)**. **(I)** UMAP projection of gene expression data. **(J)** UMAP projection of ATAC data. **(K)** Combined UMAP projection. Both gene expression and ATAC data have been integrated using Harmony integration to remove batch effect. A combined UMAP projection has been made using weighted neighbor clustering. **(L)** Per-cluster cell proportions from both vaccines. **(M)** Per-cluster cell proportions from each subject.

Supplementary Figure 2 Gene set enrichment analysis in all cells. **(A)** Cell frequencies of all cells identified in the complete dataset (**Figure 1D**). Boxplots show the median (IQR) of relative cell frequencies per vaccine. **(B-D)** Volcano plots of differentially expressed genes (DEGs) for cDC1 **(B)** pDC **(C)** and memory B cells **(D)**. **(E)** Correlation plot showing the correlation between all significantly enriched pathways and HPV-type-specific neutralizing antibody titers, with color representing the Kendall’s Tau rank. CD4+ T cells and B cells after Cervarix compared to Gardasil-4. CD14 Mono, classical CD14+ monocytes; CD16 Mono, non-classical CD16+ monocytes; NK, Natural Killer cells; ASDC, AXL+ Siglec-6+ dendritic cells; cDC1/cDC2, conventional dendritic cells type 1 or type 2; pDC, plasmacytoid dendritic cell; CD4 TEM, CD4+ T effector memory cells; CD4 TCM, CD4+ T central memory cells; Treg, regulatory T cells. CD4 CTL, CD4+ cytotoxic T cell; CD8 TEM, CD8+ T effector memory cells; CD8 TCM, CD8+ T central memory cells.

Supplementary Figure 3 Gene expression correlation with breadth of neutralizing antibody response. **(A, B)** Volcano plots of differentially expressed genes (DEGs) for dendritc cells **(A)** and B cells **(B)** of subjects with high breadth of neutralizing antibody titers (higher than median of the sum of titers of all types) compared to cells of subjects with low breadth of neutralizing antibody titers (lower than median of the sum of titers of all types). **(C)** All enriched pathways in dendritic cells and B cells. Data are stratified by subjects with a high breadth of neutralizing antibody titers (greater than the median sum of titers across all HPV types) versus those with a low breadth of neutralizing antibody titers (less than the median sum of titers across all HPV types). Positive NES indicates a pathway enriched in subjects with high breadth of neutralizing antibody titers. cDC1/cDC2, conventional dendritic cells type 1 or type 2; pDC, plasmacytoid dendritic cell; NES, normalized enrichment score.

Supplementary Figure 4 Epigenetic remodeling and chromatin accessibility in pDC. **(A)** Heatmap showing normalized chromatin accessibility at the top 200 DARs in pDC for each subject. Regions were classified as follows: promoter −2,000 bp to +500 bp; distal −10 kbp to +10 kbp – promoter; trans< −10 kbp or > +10 kbp. **(B)** Heatmap of normalized accessibility of NOTCH-related DARs in cDC1 for each subject. **(C)** Top enriched motifs identified from top 200 DARs in cDC1. **(D)** Top enriched motifs identified from NOTCH-related DARs in pDC. **(E)** Top enriched motifs identified from top 200 DARs in pDC. **(F)** Chromatin accessibility at the locus of NOTCH ligands (NOTCH1, NOTCH2, NOTCH3, NOTCH4, DLL4, JAG1) in pDC grouped per vaccine.

Supplementary Figure 5 Cell-cell communication analysis. Target gene heatmap showing the influence on expression of all inferred target genes in memory B cells (columns) by the top ligands sent by all Th cells (rows). cDC1/cDC2, conventional dendritic cells type 1 or type 2; pDC, plasmacytoid dendritic cell; CER, Cervarix; GAR, Gardasil-4.
