# Supplementary figures and images for: AS04 drives superior cross-protective antibody response by increased NOTCH signaling of dendritic cells and proliferation of memory B cells
Source: Front Immunol. 2025 Jul 24;16:1623405. doi: 10.3389/fimmu.2025.1623405 (PMC12344523; doi:10.3389/fimmu.2025.1623405)

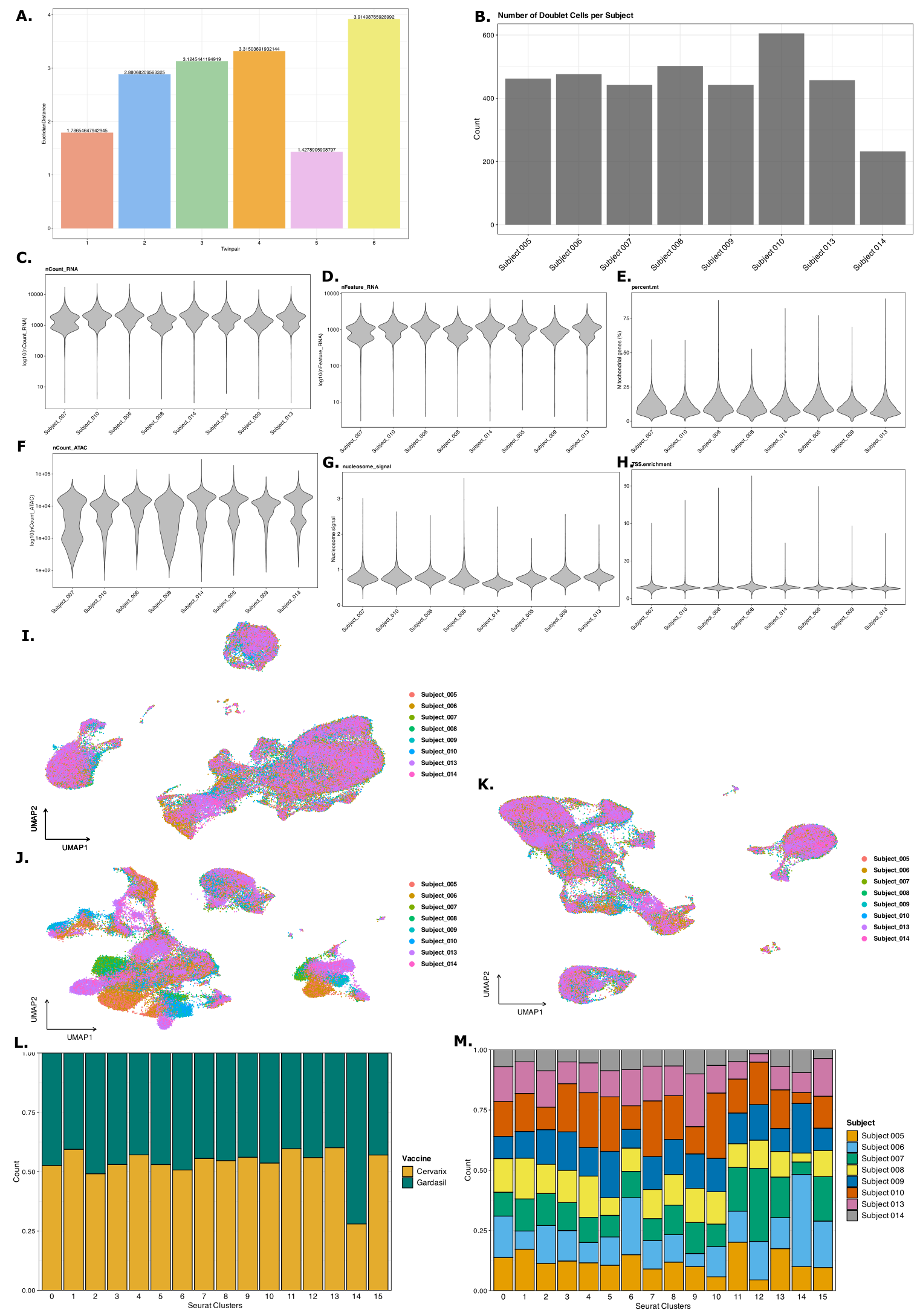

Supplement: Supplementary Figure 1 — Single cell RNA and ATAC sequencing quality. (A) Euclidian Distances between twin sisters based on neutralizing antibody titers for all HPV types. (B) Bargraph showing the number of doublet cells for each subject identified using the DoubletFinder package. (C-H) Violin plots showing the distribution of six quality metrics across all cells. Quality metrics were number of reads (C), number of transcripts (D), percentage of mitochondrial genes (E), number of ATAC reads (F), nucleosome signal (G), and TSS enrichments score (H). (I) UMAP projection of gene expression data. (J) UMAP projection of ATAC data. (K) Combined UMAP projection. Both gene expression and ATAC data have been integrated using Harmony integration to remove batch effect. A combined UMAP projection has been made using weighted neighbor clustering. (L) Per-cluster cell proportions from both vaccines. (M) Per-cluster cell proportions from each subject. [file Image1.tiff]

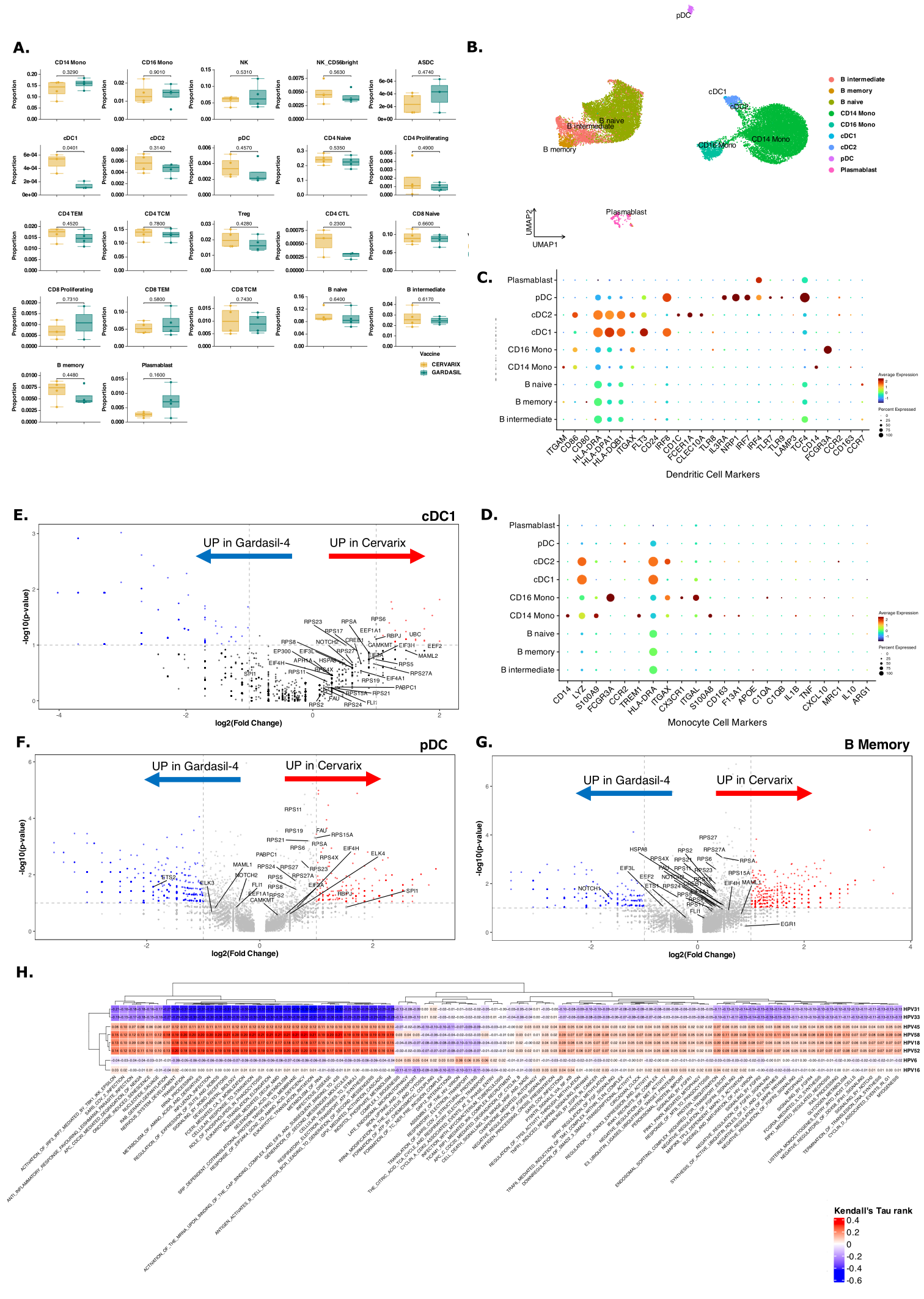

Supplement: Supplementary Figure 2 — Gene set enrichment analysis in all cells. (A) Cell frequencies of all cells identified in the complete dataset ( Figure 1D ). Boxplots show the median (IQR) of relative cell frequencies per vaccine. (B-D) Volcano plots of differentially expressed genes (DEGs) for cDC1 (B) pDC (C) and memory B cells (D). (E) Correlation plot showing the correlation between all significantly enriched pathways and HPV-type-specific neutralizing antibody titers, with color representing the Kendall’s Tau rank. CD4+ T cells and B cells after Cervarix compared to Gardasil-4. CD14 Mono, classical CD14+ monocytes; CD16 Mono, non-classical CD16+ monocytes; NK, Natural Killer cells; ASDC, AXL+ Siglec-6+ dendritic cells; cDC1/cDC2, conventional dendritic cells type 1 or type 2; pDC, plasmacytoid dendritic cell; CD4 TEM, CD4+ T effector memory cells; CD4 TCM, CD4+ T central memory cells; Treg, regulatory T cells. CD4 CTL, CD4+ cytotoxic T cell; CD8 TEM, CD8+ T effector memory cells; CD8 TCM, CD8+ T central memory cells. [file Image2.tiff]

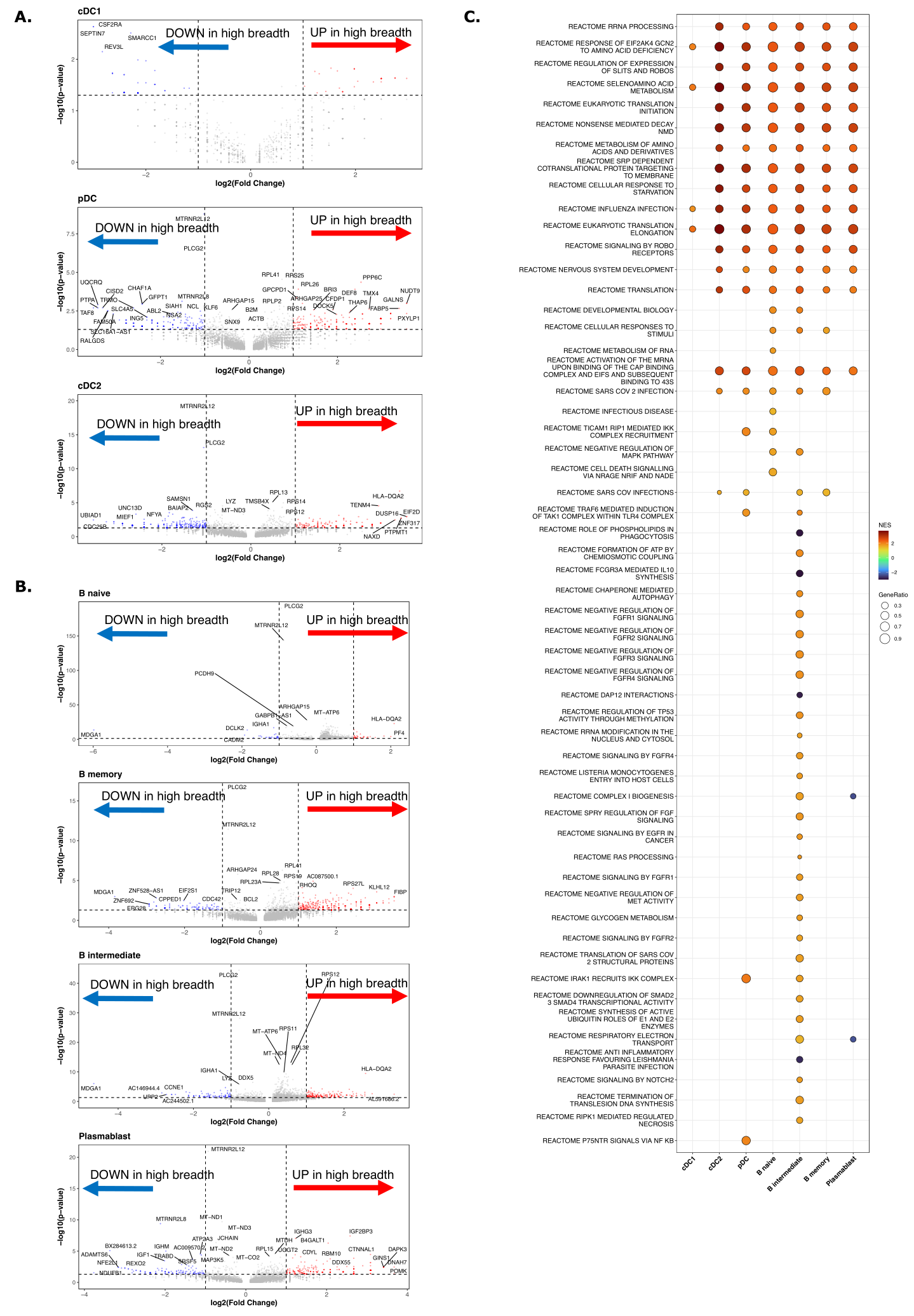

Supplement: Supplementary Figure 3 — Gene expression correlation with breadth of neutralizing antibody response. (A, B) Volcano plots of differentially expressed genes (DEGs) for dendritc cells (A) and B cells (B) of subjects with high breadth of neutralizing antibody titers (higher than median of the sum of titers of all types) compared to cells of subjects with low breadth of neutralizing antibody titers (lower than median of the sum of titers of all types). (C) All enriched pathways in dendritic cells and B cells. Data are stratified by subjects with a high breadth of neutralizing antibody titers (greater than the median sum of titers across all HPV types) versus those with a low breadth of neutralizing antibody titers (less than the median sum of titers across all HPV types). Positive NES indicates a pathway enriched in subjects with high breadth of neutralizing antibody titers. cDC1/cDC2, conventional dendritic cells type 1 or type 2; pDC, plasmacytoid dendritic cell; NES, normalized enrichment score. [file Image3.tiff]

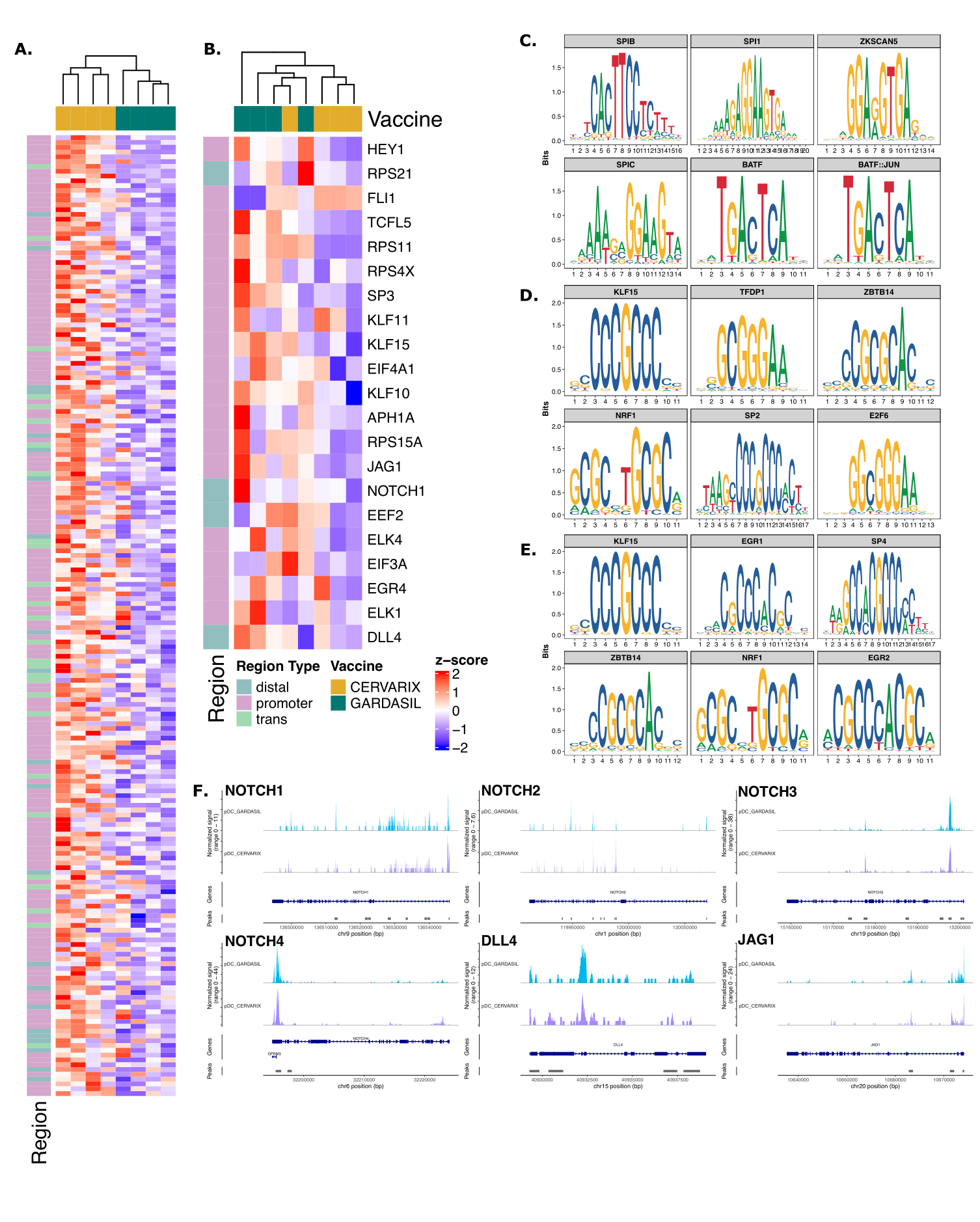

Supplement: Supplementary Figure 4 — Epigenetic remodeling and chromatin accessibility in pDC. (A) Heatmap showing normalized chromatin accessibility at the top 200 DARs in pDC for each subject. Regions were classified as follows: promoter −2,000 bp to +500 bp; distal −10 kbp to +10 kbp – promoter; trans< −10 kbp or > +10 kbp. (B) Heatmap of normalized accessibility of NOTCH-related DARs in cDC1 for each subject. (C) Top enriched motifs identified from top 200 DARs in cDC1. (D) Top enriched motifs identified from NOTCH-related DARs in pDC. (E) Top enriched motifs identified from top 200 DARs in pDC. (F) Chromatin accessibility at the locus of NOTCH ligands (NOTCH1, NOTCH2, NOTCH3, NOTCH4, DLL4, JAG1) in pDC grouped per vaccine. [file Image4.tiff]

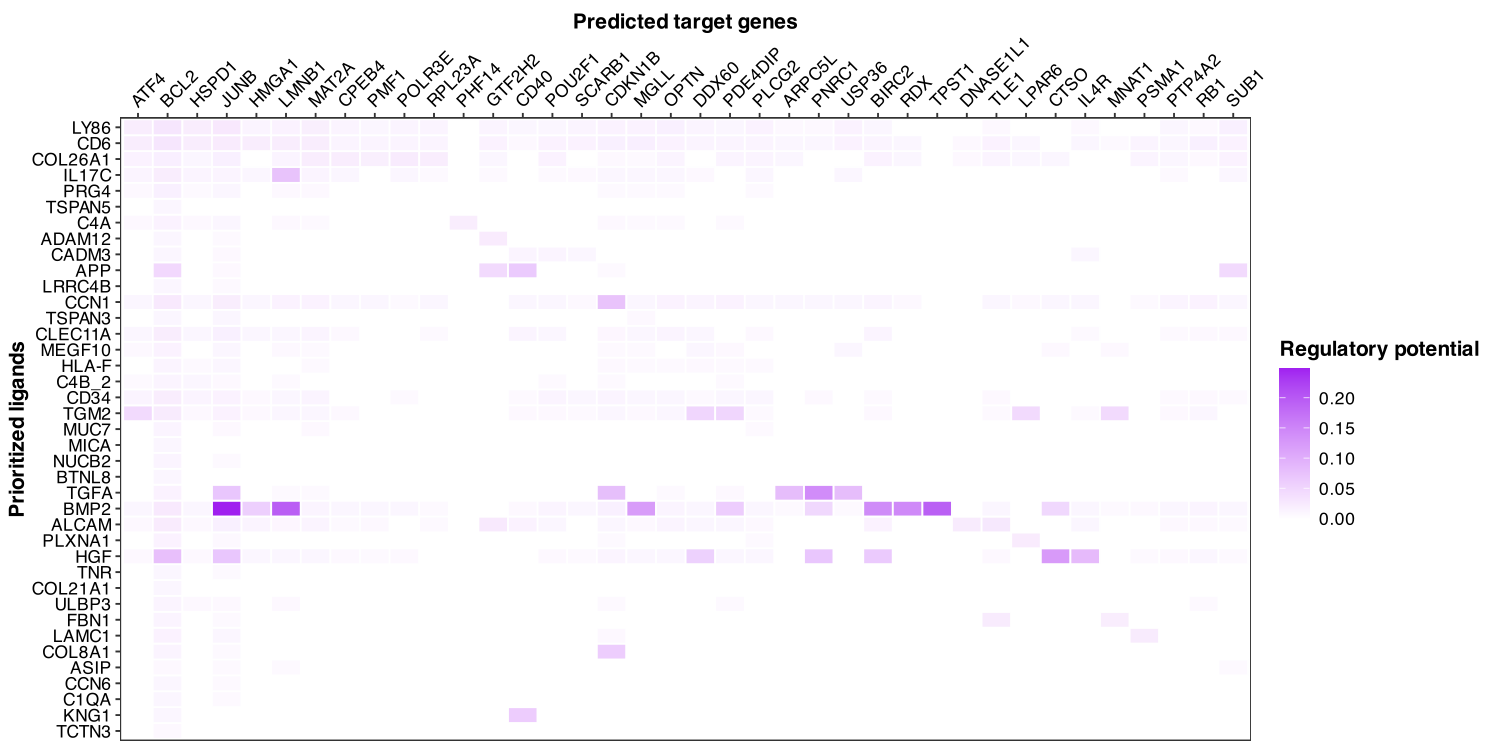

Supplement: Supplementary Figure 5 — Cell-cell communication analysis. Target gene heatmap showing the influence on expression of all inferred target genes in memory B cells (columns) by the top ligands sent by all Th cells (rows). cDC1/cDC2, conventional dendritic cells type 1 or type 2; pDC, plasmacytoid dendritic cell; CER, Cervarix; GAR, Gardasil-4. [file Image5.tiff]
